# Supplementary material for: Evidence supported by Mendelian randomization: impact on inflammatory factors in knee osteoarthritis
Source: Front Med (Lausanne). 2024 May 28;11:1382836. doi: 10.3389/fmed.2024.1382836 (PMC11165061; doi:10.3389/fmed.2024.1382836)
Supplement: Supplementary file 1 [file Data_Sheet_1.ZIP › data/GCST90274846_5e6/again/846.leaveoneout.pdf]

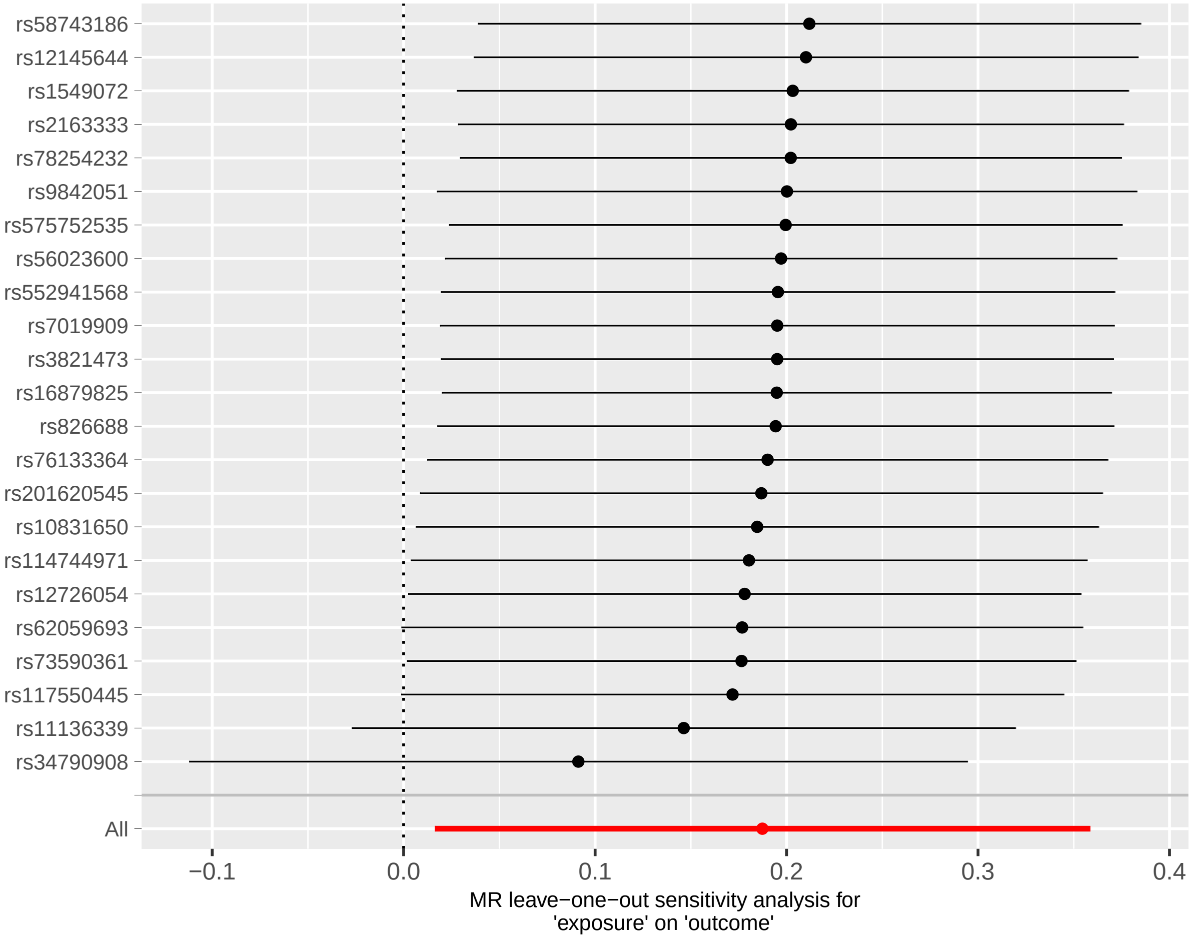
rs58743186
rs12145644
rs1549072
rs2163333
rs78254232
rs9842051
rs575752535
rs56023600
rs552941568
rs7019909
rs3821473
rs16879825
rs826688
rs76133364
rs201620545
rs10831650
rs114744971
rs12726054
rs62059693
rs73590361
rs117550445
rs11136339
rs34790908
All
−0.1
0.0
0.1
0.2
0.3
0.4
MR leave−one−out sensitivity analysis for
'exposure' on 'outcome'
